# Supplementary material for: Patterns of Intron Gain and Loss in Fungi
Source: PLoS Biol. 2004 Nov 30;2(12):e422. doi: 10.1371/journal.pbio.0020422 (PMC532390; doi:10.1371/journal.pbio.0020422)
Supplement: Table S1 — Also available at http://genes.mit.edu/NielsenEtAl/. (4.3 MB ZIP). [file pbio.0020422.st001.zip › NielsenEtAl/html/163.html]

AN5757.1.NCU04005.1.MG08097.1.FG10066.1


```
 CLUSTAL W (1.82) Multiple Sequence Alignments - Introns Inserted


Sequence 1: AN5757.1	444 aa
Sequence 2: FG10066.1	530 aa
Sequence 3: NCU04005.1	441 aa
Sequence 4: MG08097.1	534 aa
Alignment Length: 548 aa
Number Identitical Residues: 291 aa
Alignment Score (without introns) 11940


MG08097.1 	MASS-SSNVVGVHYRVGKKIGEGSFGVIFEGTNLLNNQQVAIKF0EPRKSDAPQLRDEYR
NCU04005.1	MASSTSSNVVGVHYRVGKKIGEGSFGVIFEGTNLLNNQQVAIKF0EPRKSDAPQLRDEYR
FG10066.1 	MASS-SSNVVGVHYRVGKKIGEGSFGVIFEGTNLLNNQQVAIKF0EPRKSDAPQLRDEYR
AN5757.1  	MASS-SSNVVGVHYRVGKKIGEGSFGVIFEGTNLLNNQQVAIKF0EPRKSDAPQLRDEYR
          	**** *************************************** ***************

MG08097.1 	TYKILVGCP1GIPNVYYFGQEGLHNILVIDLLGPSLEDLFDHCNRRFTIKTVVMVAKQM0
NCU04005.1	TYKILVGCP1GIPNVYYFGQEGLHNILVIDLLGPSLEDLFDHCNRRFSLKTVVMVAKQM0
FG10066.1 	TYKILVGCP1GIPNVYYFGQEGLHNILVIDLLGPSLEDLFDHCGRRFSIKTVVMVAKQM~
AN5757.1  	TYKILVGCP1GIPNVYYFGQEGLHNILVIDLLGPSLEDLFDHCNRRFSVKTVVMVAKQM0
          	********* *********************************.***::********** 

MG08097.1 	LSRVQTIHEKNLIYRDIKPDNFLIGRPGTKTANVIHVVDFGMAKQYRDPKSKQHIPYRER
NCU04005.1	LSRVQTIHEKNLIYRDIKPDNFLIGRQGTKTANVIHVVDFGMAKQYRDPKTKQHIPYRER
FG10066.1 	LSRVQTIHEKNLIYRDIKPDNFLIGRPGTKASSVIHVVDFGMAKQYRDPKTKQHIPYRER
AN5757.1  	LSRVQTIHEKNLIYRDIKPDNFLIGRPNSKAANVIHVVDFGMAKQYRDPKTKQHIPYRER
          	************************** .:*::.*****************:*********

MG08097.1 	KSLSGTARYMSINTHLGREQSRRDDLEALGHVFMYFLRGGLPWQGLKAATNKQKYEKIGE
NCU04005.1	KSLSGTARYMSINTHLGREQSRRDDLEALGHVFMYFLRGGLPWQGLKAATNKQKYEKIGE
FG10066.1 	KSLSGTARYMSINTHLGREQSRRDDLEALGHVFLYFLRGGLPWQGLKAATNKQKYEKIGE
AN5757.1  	KSLSGTARYMSINTHLGREQSRRDDLEALGHVFMYFLRGGLPWQGLKAATNKQKYEKIGE
          	*********************************:**************************

MG08097.1 	KKQTTAIKELCEGFPI~QFEKYLTYVRNLGFEDTPDYDYLRELFTQALKESGGVEDGEYD
NCU04005.1	KKQTTAIKDLCEGFPE~QFAQYLTYVRNLGFEDQPDYDYLRELFSQALKDAGGVEDGEYD
FG10066.1 	KKQTTIIKDLCEGFPE~EFSKYLTYVRNLGFEDTPDYDYLRELFTQALKNTGEVEDGEYD
AN5757.1  	KKQTTAIKELCEGFPE1EFTKYLSYVRNLGFEDTPDYDYLRDLLTQALKNAGEVEDGEYD
          	***** **:******  :* :**:********* *******:*::****::* *******

MG08097.1 	WMKVSK-DKKNDWDR------QAMHNPSQRPGASNMELHGASRGAHASDLRDPRARLTVD
NCU04005.1	WMKVNKSDKKGDWDRP-----GALHNPNARPGPSAMEIH-NSRGPTTHHASDPRAQLTAA
FG10066.1 	WMKISKDSGKG-WDSKNHSN-AYLHNPNVRPGPSQMELHSGHRPGNTTSHQQ-AQNLTVG
AN5757.1  	WMKLN--NGRG-WEYKSYSSQQALQN-------SAMHTSARDLHGQQIRNSQ-RPGVTAD
          	***:.  . :. *:  . :..  ::*       * *.  .           :    :*. 

MG08097.1 	RLNAAQPPPPSPIKANQGKDR--RNAPGAIMASRGT-AGGLRDMATPTGSTQAQFQNSTQ
NCU04005.1	RLNAPQPSPP----AQPGQRA--KEQPNANAQNN----RAYKSQPTPTQSAGNGAQPYNM
FG10066.1 	RLNAAQPPPPSPIKQ-MGKQRDRPSAPGALSAQRGSGVGGLRDMATPTGSTQAQFQNSAQ
AN5757.1  	RLNAAQPPPPSPAKPGAGKTREPQNVRGGIPPKRQS--GGFD--TTPAASTVAQFQNSNA
          	****.**.**:. .   *:  .  .  ..   .. :   .    .**: *:    *    

MG08097.1 	NLPQRPMH-QQSNMTQPQLNNGRPAEP--EPTGFQKFMK0LRRS~A~SSQRSTVGPTLWE
NCU04005.1	QQPP------QRTMTHTATSNTNPPP-------------~----~-~-------------
FG10066.1 	NLPQQPRTSQQGPATQPTQASGQPAN--PQPSGFQKLMK~TLCF1V~RVRN------LSL
AN5757.1  	NISGHRIGSPATPAKNQPAPGGQQASNEPQPTLMQKVMK~ALCC~G1-------------
          	: .     :     .:    . . .  ....:  ..  .                     

MG08097.1 	TCTNISGAPAVFSSQPNARLASNMRYSRGFTGTIAPCRATSLRRIADMLNVDDDGTGTKP
NCU04005.1	------------SNQPQQQQGG-------------------IKGIWKALCCS--------
FG10066.1 	SCSPRCYCSPERQSHHARTRSPSSRYFRTLPPPSHPIFSCMTTREGKQIWFGITAKGFPF
AN5757.1  	------------------------------------------------------------
          	                                                            

MG08097.1 	FHHHNHPLLRRLRKK
NCU04005.1	---------------
FG10066.1 	SCLTTITKLY-----
AN5757.1  	---------------
          	
```
